# La Vida Buena (The Good Life) evaluation: a quasi experimental intervention of a community health worker-led family-based childhood obesity program for Latino children 5–8 years of age on the US-Mexico border

**DOI:** 10.1186/s12889-019-7081-x

**Published:** 2019-06-14

**Authors:** Kathryn M. Tucker, Maia Ingram, Kevin Doubleday, Rosie Piper, Scott C. Carvajal

**Affiliations:** 1 University of Arizona Prevention Research Center, 1295 N Martin Ave, Tucson, AZ USA; 2 Mariposa Community Health Center, 1710 N Mastick Way, Nogales, AZ USA

**Keywords:** Community health workers, Childhood obesity, Community-academic partnership, Latino

## Abstract

**Background:**

Due to multiple and interacting factors, Latino children are disproportionately at risk for overweight and obesity in the United States. Childhood obesity increases the risk for adverse physical and psychosocial outcomes throughout the lifespan. Intensive behavioral interventions recommended in primary care settings may not conform to current practices, and the most vulnerable populations are often unable to access these services. Community Health Workers (CHWs) off**e**r a promising approach to bridging the gap between vulnerable communities and culturally competent services. La Vida Buena (The Good Life) is an 8-week family-focused intervention for Latino children 5–8 years old and their parents or caregivers who are patients at a Federally-Qualified Community Health Center (FQHC). It is a culturally and linguistically appropriate curriculum, facilitated by CHWs, that targets family behaviors to foster a healthy lifestyle in order to prevent and mitigate childhood overweight and obesity.

**Methods:**

The primary objective is to test the effectiveness of the La Vida Buena (LVB) childhood obesity program among Latino children 5–8 years old and their families as compared with a single educational session. This study uses a parallel two-arm quasi-experimental design. The intervention group receives the 8-week La Vida Buena intervention and the comparison group receives a single educational session. The primary outcome is the change in the child’s BMI z-score from baseline to 6 months.

**Discussion:**

The implementation and evaluation of La Vida Buena may inform research and practice for linking Latino patients in FQHCs to culturally responsive community-based childhood obesity interventions. It will also contribute to the literature about CHWs as facilitators of behavior change for families underserved by health services and preventive programs. La Vida Buena can serve as a culturally and linguistically appropriate early intervention curriculum that will foster a healthy home environment for childhood obesity mitigation and prevention.

**Trial registration:**

The trial was retrospectively registered on December 18, 2018. The ClinicalTrials.gov Identifier is NCT03781856.

## Background

The Latino population bears a significant burden of overweight and obesity in the United States. The prevalence of obesity in Latino adults is 47%, as compared with 39.8% for adults overall and 37.9% of non-Hispanic white adults. This trend appears to be initiated in childhood; the prevalence of obesity among Latino youth is 25.8%. as compared with 18.5% overall, and 14.1% for non-Hispanic white youth [1].

Childhood obesity is associated with adverse physical and psychosocial outcomes that carry into adulthood. Childhood and adolescent overweight and obesity are associated with poorer health status, lower emotional functioning, and worse educational performance [2–4]. Children with obesity are more likely to experience psychosocial issues such as anxiety, depression, low self-esteem and bullying [3, 5]. Children with obesity are likely to become adults with obesity [6], and their health issues tend to be more severe as adults [7–9]. Obesity is associated with a variety of serious health conditions, including hypertension, type 2 diabetes, heart disease, stroke and cancer [8–10].

Importantly, the major risk factors for childhood overweight and obesity include modifiable environmental and behavioral factors. Family behaviors such as eating calorie dense and nutrient-poor foods, drinking sugar-sweetened beverages, not getting enough physical activity, not eating together, not sleeping enough and spending too much time doing sedentary activities all contribute to excess weight gain [9]. For Latino families in particular, home environment factors such as parental influences, screen time, sedentary activity, food security and sleep duration are associated with childhood obesity [11]. Broader environmental and policy factors such as the affordability and accessibility of healthy foods, marketing and promotion, community design, school environment and availability of safe physical activity spaces further shape individual and family behaviors that impact obesity [12].

The CDC defines childhood overweight as a BMI at or above the 85th percentile for children of the same age and sex, and obesity as a BMI at or above the 95th percentile [7]. The US Preventive Service Task Force recommends that clinicians measure children’s height and weight and refer children with overweight or obesity to comprehensive, intensive behavioral interventions [13]. Successful interventions include over 26 contact hours, family involvement, and multidisciplinary facilitation teams composed of clinicians, dietitians and social workers [13]. However, these recommendations are misaligned with current practices in many primary care environments, and they do not necessarily reach the populations most in need of intervention [14]. A systematic review of recruitment and retention in obesity prevention trials targeting minority or low-income children found that retention rates were lower for trials that only targeted Latinos, involved children and parents, focused on overweight or obese children, were community-based, or included both nutrition and physical activity [15].

Community Health Workers (CHWs), frontline public health workers with a unique connection to the community served, offer a promising approach to engage and execute health promotion efforts with Latino populations [16].CHWs can serve as culturally competent mediators between communities and health service providers. Numerous studies have documented the effectiveness of community-based CHW programs in improving maternal and child health outcomes [17–19] as well promoting the prevention and management of chronic diseases [20, 21].

There is a small but growing body of research exploring the effectiveness of CHW-led childhood obesity programs. CHW-led childhood obesity programs have been implemented in home settings [22, 23], through schools [24] and at an urban health center [25]. These interventions demonstrate that a culturally competent program facilitated by a CHW can be effective in promoting healthy diet and physical activity habits in Latino populations.

This study protocol describes La Vida Buena (The Good Life), a childhood obesity intervention developed by an FQHC in a rural county along the US-Mexico border. The 8-week family-focused program, co-developed and facilitated by CHWs, aims to address childhood obesity in Latino children 5–8 years old and their families who are patients at the Federally Qualified Community Health Center (FQHC). The curriculum is guided by the socio-ecological framework [10, 26] and focuses on behavior change to foster healthy home environments and connection to community resources. The intervention includes activities related to physical activity and nutrition aimed at fostering healthier family habits.

### Objectives

The research study’s primary objective is to test the effectiveness of the 8-week, CHW-led La Vida Buena childhood obesity program among Latino children 5–8 years old and their families as compared with a single educational session.

### Research design and setting

#### Trial design

This is a parallel two-arm quasi-experimental behavioral trial based in two communities.

#### Study setting

The study takes place in two communities in a low-income, rural county along the US-Mexico border. The majority (83%) of residents are Latino, primarily of Mexican origin. Nearly a quarter (24%) of residents live below 100% of the federal poverty level and over a third (35%) of children live in poverty [27, 28] A third of residents (35%) are foreign-born and 81% use a language other than English at home. The county is designated as a Health Professional Shortage Area by the Health Resources and Services Administration [29].

The FQHC that developed and is leading the intervention is the medical home for 91% of all children aged 0–19 in the county, and acts as the county health department. Thirty percent of the FQHC’s pediatric patients have a documented BMI greater than the 85th percentile, exceeding the threshold for childhood overweight/obesity. The Community Health Services Department at the FQHC developed the La Vida Buena curriculum, which is offered offsite in a community setting.

#### Justification for study comparators and design

The study population includes patients from two of the FQHC’s four clinics: the comparison group is drawn from patients in a satellite clinic 10 miles from the intervention community. The patient populations of the two communities are demographically similar. After outcome data collection is complete, the comparison group will have the opportunity to participate in the full intervention.

Our rationale for a quasi-experimental study design takes into account the realities of an FQHC in a rural setting. Randomization would be awkward with a project in which pediatricians are referring their patients, and it would be uncomfortable for CHWs and medical staff to explain and justify random assignment to parents. Group-based randomization was not possible because the FQHC has too few clinics. The use of a comparison group from a different clinic site with a similar demographic profile is a practice-based research design that affords key evidence for other FQHCs that wish to adopt and evaluate the intervention [30].

#### CBPR approach

This study uses a Community-Based Participatory Research (CBPR) approach fostered by over 20 years of academic-community partnership between the FQHC and their academic partners at a nearby university [31]. CBPR is the co-construction of research through partnerships between researchers and the people affected by, or responsible for, action on the issue under study. Some of the benefits of participatory approaches include the creation of culturally and logistically appropriate research, enhanced recruitment, increased stakeholder capacity, improved sustainability of project goals, and greater potential for systems change [32, 33].

The collaborative and participatory evaluation methods in La Vida Buena includes engagement of a community advisory board (CAB) and a participant advisory board (PAB). The CAB provides support for project development, resources, recruitment and intervention activities. The CAB includes partners from organizations throughout the county, including a biking nonprofit, representatives from local schools, the cooperative extension, and the local farmer’s market. The participant advisory group (PAB) is comprised of parents who have completed the program and who provide the perspective of parents with young children in project planning and dissemination.

Both the CAB and the PAB are active in community- and systems-change activities to complement the emphasis on family behavior change in La Vida Buena. It is essential to integrate clinical and community systems to address childhood obesity [16]. The La Vida Buena team works with the CAB and PAB to identify systems and policy changes that foster a healthy environment in the county.

### La Vida Buena intervention

#### Intervention development

La Vida Buena was adapted from a program that the FQHC originally delivered to adolescents 10–19 years old; parents were invited but not required to participate. An initial pilot adoption and preliminary evaluation of the program using pre- and post-tests showed statistically significant improvement in nutrition-related health behaviors, including decreased consumption of sugar-sweetened beverages (SSBs) and fast food consumption and increased fruit and vegetable consumption.

In the current La Vida Buena Program, the FQHC adapted the program for younger children in order to intervene at an earlier age and implement a more rigorous evaluation framework. The FQHC leadership decided to implement the intervention with a younger population because the proportion of children with overweight and obesity increases steadily through the childhood and teenage years [34, 35]. Early intervention is critical to prevent the physical and psychosocial effects of overweight and obesity. This program focuses on a family-based strategy as a way to shape behaviors in the home, which are key risk factors for developing overweight and obesity [36]. LVB is designed to target key family behaviors that will shape the child’s diet and physical activity habits at a crucial time.

The LVB team used the structure of the original program to design a curriculum geared towards children 5–8 and their parents. The FQHC piloted the curriculum and refined the intervention based on feedback from the CHWs, observers and participants. Revisions included ensuring that the language was regionally appropriate, that activities engaged both children and parents, that the discussion included both parents and children, and that physical activity was age-appropriate.

In the curriculum design, the FQHC and academic partner carefully considered language and framing. While we recognize that childhood overweight and obesity has serious health implications that last throughout the life span, the La Vida Buena program focuses on a healthy diet, behavior change for a healthy home environment, and positive aspects of a healthy lifestyle, rather than weight or the negative consequences of overweight and obesity.

##### La Vida Buena intervention

The LVB team includes two CHWs, a CHW coordinator, a health promotion manager at the FQHC, two evaluators based out of a nearby university, and two adolescent peer educators. The La Vida Buena intervention, delivered by teams of adult Community Health Workers (CHWs) and adolescent peer health educators (Teen Health Facilitators), takes place once a week for 8 weeks. Each weekly hour-long session includes 20 min of interactive learning topics, 20 min of physical activity and 10 min of healthy food preparation and discussion. All activities are designed to encourage active learning from both the parents and the children. The interactive learning topics focus on nutrition, physical activity and healthy family habits. Teen health facilitators lead the physical activity portion of the class. The objectives of each class session and selected activities are listed in Table 1.

**Table 1:** La Vida Buena curriculum session objectives

Session Name	Objectives	Example activities, incentives and food demonstrations
Session 1: Orientation	• Identify the program goals: Increase fruit and vegetable consumption; Increase non-school physical activity; Reduce sugary drink intake and other refined carbohydrates • Identify two health issues related to being overweight • Recognize La Vida Buena as an informative and fun program	• Physical activity through play • Incentive: jump ropes • Food demo: fruit salad
Session 2: Gotta Love those Fruits and Veggies	• Name GO FOODS - to eat almost anytime (Fruits and veggies) • Identify SLOW FOODS - to eat less often (Grains, dairy, protein) • Name WHOA FOODS - to eat once in a while (High caloric, high fat foods) • Express one strategy to eat in a more healthy manner	• Go/Slow/Whoa traffic light game • Incentive: fruit/veggie stuffed animals and measuring cups • Food demo: DIY salad bar
Session 3: Guess what’s in the bowl	• Understand what a serving size is • Understand MyPlate	• Guess how much is in the cereal bowl • Incentive: MyPlate plate, smoothie cup • Food demo: cauliflower rice
Session 4: Breakfast for your Brain	• Identify breakfast as the most important meal of the day • Identify a healthy cereal by label reading based on fiber and sugar contents • Write one healthy breakfast menu as a family	• Label detective with magnifying glasses • Incentive: balls and Tupperware set • Food demo: apple “cookies”
Session 5: Physical Activity with 0S3 biking group	• Identify the benefits of bicycling • Identify opportunities to use their bikes • Name two safety rules when bicycling	• Biking activity circuit • Incentives: bike helmets • Food demo: Fruit kebab stick
Session 6: Water Your Way	• Read beverage nutrition facts labels • Name one benefit of drinking water and skim milk • Learn how much sugar is in popular drinks	• How much sugar do you pour into your bodies? • Incentives: water bottles • Food demo: infused water
Session 7: Shopping Like Pros	• Learn to navigate the supermarket • Identify strategies to be prepared for grocery shopping • Read nutrition facts label	• Choose a meal from the La Vida Buena ‘supermarket’ • Incentives: shopping list and chef hat • Food demo: avocado toast
Session 8: My Mexican Food is Healthy	• Identify one strategy to cook healthier • Modify one recipe by adding, reducing, substituting, or eliminating to create a healthier recipe • Express one benefit of eating together	• Make abuelita’s recipe healthier • Incentives: crock pot • Food demo: yogurt parfait

Culturally responsive educational tools and incentives are intended to maximize participation, reinforce learning objectives and increase participant retention. Teaching tools include MyPlate plates, plastic foods, posters, and magnifying glasses to ‘investigate’ labels, among other items. Incentives include items such as jump ropes, cooking utensils, cookbooks and water bottles.

#### Comparison group

The comparison group is drawn from a satellite clinic in a neighboring community. The characteristics of the pediatric population are similar in both clinics. CHWs deliver a 50-min educational session either one-on-one or in a group setting. The educational session includes 40 min of interactive learning activities focused on nutrition and 10 min of physical activity information, and focuses on some of the basic strategies and key messages covered in the 8-week La Vida Buena intervention. The CHW facilitator discusses nutrition and physical activity strategies with the parent and child in a single educational session, but the session is more didactic and does not include a physical activity session. The session objectives for the comparison group are listed in Table 2.

**Table 2:** Learning Objectives for Comparison Group Single Educational Session

Session	Objectives
Comparison Group Education Session	By the end of the session participants will be able to: • Identify Go foods - to eat almost anytime (Fruits and Veggies) • Identify Slow foods - to eat sometimes (Grains, Dairy, Protein) • Identify Whoa foods - to eat once in a while (High caloric, High fat foods) • Identify the recommended daily fruit and vegetables portions

The FQHC will offer the full LVB intervention to participants in the comparison group following the completion of data collection.

#### Intervention Fidelity

An academic evaluator and the LVB coordinator established definitions and expectations for intervention fidelity using a participatory process. They observed and rated the delivery of selected sessions using a fidelity checklist based on session objectives, facilitator skills, and participant responses. The pair iteratively compared and resolved rating differences until they had established 80% inter-rater agreement in every session. For the remainder of the intervention and comparison sessions a single observer attends and evaluates at least 20% of the sessions, using a 5-point Likert scale to assess intervention delivery and response. Fidelity is considered an average of 4.0 or above for each scale.

## Methods: recruitment

### Sample size

To detect a minimal important difference of 0.2 BMI z-standardized units between baseline and 6-month follow-up, an estimated sample size of *n* = 200 participants is required. This calculation assumes 100 participants for the intervention site and 100 participants for the comparison site, with a 25% attrition rate, in order to achieve 80% power. The power calculation was completed using ANCOVA simulations with a type 1 error rate of 5% and a standard deviation in group differences of 0.4 BMI z standardized units.

### Eligibility criteria

Study inclusion criteria include: [1] age of child (5–8 years old), [2] BMI of child above 85th percentile for age and sex, [3] participation of an adult parent or caregiver who is 18 or older, and [4] signed consent from the parent or guardian. All intervention activities are completed in Spanish, so both the parent and child must speak and understand Spanish.

### Recruitment and retention

Participants are recruited through the FQHC clinics. Medical providers refer patients who are eligible for the program based on the inclusion criteria. Medical assistants, care coordinators, and CHWs work collaboratively to invite participants at doctor’s appointments, or by printed invitations, telephone calls and/or the patient portal. The LVB study team also created flyers and brochures that can be provided to potential participants. The CHWs who coordinate and facilitate the program call to confirm their participation and begin to build relationships with the participants before the first class.

Based on an intent to treat analysis, once enrolled in the program, each family is considered a participant for post and follow up measure, regardless of the extent to which they engage in La Vida Buena curriculum. Study staff remind participants of the weekly activities and keep in touch with participants throughout the program through phone calls and text messages. The FQHC has identified strategies, such as the incentives, to keep participants engaged in the La Vida Buena program activities. We also offer childcare and transportation to the families to reduce potential barriers to attending the class. Parent participants are provided with a $25 gift certificate to a local grocery store upon completion of both the post and follow-up measures.

## Data collection, management and analyses

### Outcome data collection

CHWs collect child anthropomorphic measures and parent self-report data from participants at three time points: baseline, 3 months (1 month post-intervention) and 6 months (4 months post-intervention). CHWs collect child anthropomorphic measures with a portable stadiometer and scale. Measurements for both weight and height are taken twice. If the two measurements differ by more than 0.5 cm for height and 0.5 lbs. for weight, a third measurement is taken. The final measurement is the average of the two measurements that coincide most.

The questionnaires on family behavior and child nutrition and physical activity is administered by trained CHWs using REDCap [37]. The information is either collected on iPads or on printed copies of the questionnaire and transcribed later.

### Qualitative data collection

The LVB evaluation uses mixed methods, with qualitative data complementing quantitative outcomes to contextualize the results. The CHWs conduct focus groups with a sample of the parent participants immediately after the intervention, 1 month after the intervention or 4 months after the intervention. The focus groups correspond with the post and follow-up data collection time points. The focus group explores process-oriented themes to help the LVB team understand barriers to attendance, and support recruitment efforts. The focus groups also explore themes of motivation, making and maintaining healthy family behavior changes, and the physical activity and nutrition environment in the intervention community.

While the parents complete the focus group, the children participants also take part in guided evaluation activities. The activities are modeled after the interactive and playful format of the class, with age-appropriate games and drawings. The guided activities explore the children’s perceptions of health and what they learned from the class. The teen health facilitators, aided by a CHW or the university evaluator observer, facilitate the guided activities with children.

### Data management

REDCap is a secure web application supported by the University of Arizona that allows online data entry and management [37]. REDCap complies with the Health Insurance Portability and Accountability Act (HIPAA) security guidelines and is approved and endorsed by the University of Arizona Privacy Office and the Institutional Review Board (IRB).

The LVB coordinator or evaluator check the REDCap data after each data collection to verify accuracy and quickly correct any potential issues. There are also quality control measures built into REDCap, which display the height and weight from the previous data collection point for the post and follow-up measurements so that the CHWs can easily check the measurements for errors. In the event that the LVB team is unsure about the accuracy of child anthropomorphic measurements, the CHWs will seek written consent from participants to check the values against height and weight measurements recorded in the FQHC’s Electronic Health Record around the same time period.

### Outcomes

#### Primary outcomes

The primary outcome is the change in BMI z-score between baseline and 6 months follow up among subjects participating in the program La Vida Buena as compared with those receiving a single educational class.

#### Secondary outcomes

Secondary outcomes include difference in family-focused healthy lifestyle practices, difference in fruit and vegetable consumption, difference in the consumption of sugary drinks and beverages and other refined carbohydrates, and difference in non-school weekly physical activity between baseline and follow-up. Secondary outcomes are measured through parent self-report using the Family Nutrition and Physical Activity (FNPA) Tool and the International Physical Activity Questionnaire (IPAQ). The FNPA has been validated as a tool to capture important elements of the family environment that relate to risk for child overweight and increasing BMI [38, 39]. The International Physical Activity Questionnaire also has demonstrated validity and reliability in various settings [40]. The outcome of physical activity will be converted to youth metabolic equivalents (MET_y_ youth) units using the Youth Compendium of Physical Activity (YCPA) compiled by the National Collaborative on Childhood Obesity Research [41]. This compendium provides MET estimates for common physical activities stratified by sex and age group. The questions regarding vigorous physical activity include as examples soccer, running, and swimming. The questions regarding moderate physical activity include as example walking, skating, and paddling in a pool. The YCPA includes all these activities except skating, but rollerblading is included and can be used as a substitute for skating. Using the indicated activity, duration of the activity, and basal metabolic rate (BMR) as calculated by the Schofield equations an estimate of MET_y_ can be obtained for each participant. Note that the Schofield equations for BMR are included in the YCPA. Fruit and vegetable consumption and sugary drink consumption outcomes will be measured in units of “servings per week”.

### Statistical methods

We will summarize all baseline continuous variables using descriptive statistics, i.e. mean and standard deviation, stratified by treatment group. We will summarize categorical variables using frequencies and percentages. Baseline clinical and demographic data will be compared between the intervention and wait-list control groups. Dichotomous and ordinal variables will be examined using either chi-square test or Fishers exact test and continuous measures with two-sample t-test or two sample non-parametric Wilcoxon Rank Sum Test. Feasibility outcomes (recruitment, retention, etc.), will be described using frequencies/percentages, and 95% confidence intervals.

#### Primary analysis

The primary endpoint will be change BMI z-score from baseline to 6 months follow up among subjects participating in the program La Vida Buena compared to those receiving an educational class. BMI z-scores will be modeled using an analysis of covariance (ANCOVA) model. Let BMI_*ijk*_ represent the BMI z-score for the *j*^*th*^ subject in the *i*^*th*^ treatment group at the *k*^*th*^ time point for *k* ∈ {0, 1, 2}. The *k* levels for time point correspond to baseline (k = 0), 3 months follow up (k = 1), and 6 months follow up (k = 2). The ANCOVA model is specified as,

$$ {\mathrm{BMI}}_{ij2}={\mathrm{BMI}}_{ij0}+{T}_i+{\epsilon}_{ij2} $$

where *T*_*i*_ is the intervention effect for and *ϵ*_*ij*2_ is the error term. The effect *T*_*i*_ will be tested at the *α* = 0.05 significance level and the effect size along with a 95% confidence interval will be reported.

#### Secondary analysis

Secondary outcomes of [1] family practices, [2] physical activity, [3] consumption of fruits and vegetables, and [4] consumption of sugary drinks and other refined carbohydrates will be analyzed in a using ANCOVA models as specified for the primary outcome, i.e. secondary outcome measure at 6 months follow up is the response, intervention group is the factor to be tested, and an adjustment is made for baseline value of the outcome.

### Qualitative data analysis

We will use NVivo Software to analyze and code open-ended questions from the parent focus groups and child activities. Using a content analysis, we will identify themes related to the parent and child motivation, internalization of health information, behavior change processes and experience in the class.

### Ethics, consent and permissions

The LVB study team has obtained approval from the University of Arizona Institutional Review board for all aspects of this study (IRB Protocol Number 1710977008, approved November 15, 2017). Due to the iterative and participatory nature of this study, we submit regular amendments to ensure that our consent forms, questionnaires, procedures and protocols are appropriate. Most changes are based on feedback from the CHWs, LVB project team, and project participants.

### Informed consent

Eligible children and their parents are invited to attend the LVB orientation session. During the orientation session, each parent or caregiver participates in the informed consent process. Eligible participants provide written informed consent before being enrolled in the study. Every parent participant is provided with a hard copy of the informed consent document in Spanish or English, and their signature is collected electronically through REDCap. IRB-approved and trained research staff conduct the informed consent procedures with potential participants. After the parent participant has successfully completed the informed consent process, study staff gather baseline data consisting of anthropomorphic measures and parent self-report of the child’s behaviors related to nutrition and physical activity.

### Confidentiality

The study documents are electronically stored on password-protected computers and tablets on encrypted databases on a secure UA server. The computers, tablets and paper copies of the survey are kept in locked cabinets when not in use by the LVB research team.

### Dissemination

After completion of the LVB study, we will disseminate the results in several ways: [1] peer-reviewed publications in targeted journals; [2] presentations at scientific and practice conferences; [3] informational meetings with community members and leaders the intervention and comparison community; [4] web-based dissemination of the LVB toolkit including curriculum materials and additional resources. We will also leverage current partnerships to disseminate findings through local academic and community networks and newsletters, local media, FQCHCs, partner agencies, and local and national public health organizations. These dissemination activities will strengthen the FQHC’s ability to conduct research and disseminate findings as we identify ways to sustain the intervention activities and impact.

## Discussion

La Vida Buena will add to the growing body of research on childhood obesity interventions facilitated by CHWs in a community setting. In addition, it will provide evidence for a culturally relevant and linguistically appropriate intervention for Latino families, a population that is traditionally difficult to reach with childhood obesity interventions [15]. The LVB study is an example of a community agency implementing practice-based research in a community-academic partnership. The methods for the intervention and the research were developed in a community setting, which increases the potential for application and translation in similar community settings.

The FQHC is implementing LVB with community partners and engaging community members and participants with the support of the community advisory board and participant advisory board. The LVB team consults with the CAB and PAB regarding recruitment strategies, community resources that can be leveraged to facilitate the nutrition and physical activity practices taught in the intervention, and potential avenues for uptake and dissemination. This participatory engagement process increases the likelihood that the community will benefit from the study because the intervention is more likely to be sustained and available to the broader community if it is proven effective. This process will also engage organizations and community members in the process of identifying and fostering improvements in the community that will support and foster a healthy lifestyle for families with young children. The participatory evaluation methods improve the possibility that program activities can be sustained beyond the study and grant period.

## Data Availability

Project data sets will be housed electronically on University of Arizona servers indefinitely. The FQHC staff will have access to cleaned and de-identified data sets. Data is available upon request, after consultation with community partners.

## Abbreviations

CAB: Community Advisory Board
CHW: Community Health Worker
FQHC: Federally-Qualified Health Center
LVB: La Vida Buena (The Good Life) Program
PAB: Participant Advisory Board
